# Atrial fibrillation ablation in a single atrium with inferior vena cava interruption

**DOI:** 10.1111/anec.13057

**Published:** 2023-03-31

**Authors:** Sok‐Sithikun Bun, Fabien Squara, Didier Scarlatti, Pamela Moceri, Emile Ferrari

**Affiliations:** ^1^ Cardiology Department Pasteur University Hospital Nice France

**Keywords:** atrial fibrillation ablation, atrial septal defect, congenital heart disease, single atrium

## Abstract

Common atrium (CA), also called three‐chambered heart, is one of the rare congenital anomalies, defined by a complete absence of the atrial septum, eventually associated with malformation of the atrioventricular (AV) valves. We report the case of a 57‐year‐old woman with CA complicated with Eisenmenger syndrome and inferior vena cava interruption, who suffered from symptomatic persistent atrial fibrillation (AF). She underwent an initial successful pulmonary vein isolation procedure. A repeat procedure for perivalvular atrial flutter was complicated with inadvertent complete AV block, due to unusual AV node location in this challenging anatomy.

## Graphical abstract

We report the case of a 57‐year‐old female patient with single atrium (SA) complicated with Eisenmenger syndrome, who was referred for highly‐symptomatic persistent atrial fibrillation (AF). The patient was treated with Furosemide, Spironolactone, Rivaroxaban, Bosentan and Tadalafil. Because of symptomatic recurrences under Amiodarone after initial successful electrical cardioversion, pulmonary vein isolation (PVI) was decided. The patient was in sinus rhythm at the beginning of the procedure. In the presence of known associated inferior vena cava (IVC) interruption, the procedure was planned via a superior approach. Under mild sedation, an ultrasound‐guided puncture was performed and three catheters were inserted via the left internal jugular vein: a decapolar catheter was inserted in the coronary sinus, a multipolar catheter (PentaRay®, Biosense Webster), and a 3.5 mm irrigated contact‐force‐sensing ablation catheter (SmartTouch ST‐SF®, Biosense‐Webster Inc., Diamond Bar, CA, USA). After three‐dimensional geometry SA reconstruction (Carto System®, Biosense Webster Inc.), point‐by‐point RF delivery was performed. Target values of ablation index were 450 to 550 at the anterior wall, ≥350 on the roof and the posterior wall. The lesions set was completed by an additional posterior line with complete PVI achieved (Figure, panel A). On day 3, the patient developed “typical‐like” clockwise atrial flutter (AFL) with prolonged QTc interval (600 ms), leading to Amiodarone interruption, then switched to Acebutalol. Because of persistent symptomatic AFL (cycle length 270 ms), another ablation procedure was performed one month later. Activation mapping showed a perivalvular circuit that was interrupted by creating a line between the “blind” IVC and the valve (Video S1). During radiofrequency delivery, an inadvertent and complete atrioventricular (AV) block occurred (Figure, panel B) with junctional escape at 50 beats per minute, without any His potential recorded during high‐density mapping. A dual‐chamber pacemaker had to be implanted. After one‐year of follow‐up, the patient was free from any atrial arrhythmia recurrence without anti‐arrhythmic drug therapy. SA, also called three‐chambered heart, is one of the rare congenital anomalies, defined by a complete absence of the atrial septum, eventually associated with malformation of the AV valves. Only three previous case reports described the presence of AF in patients with SA. The eldest patient to date with SA, was a 60‐year‐old female patient with permanent AF (Rawala et al., 2019). In two recent cases, AF was the first manifestation revealing SA (Altıntaş et al., 2017; Kim et al., 2016). To the best of our knowledge, this is the first description of PVI in a patient with SA and IVC interruption. Special considerations should be taken when performing an ablation procedure, especially concerning the likelihood of atypical location of the AV node.

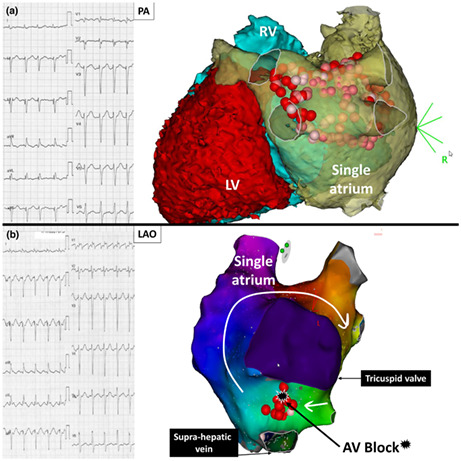

## 1 CASE PRESENTATION

Common atrium (CA), also called three‐chambered heart or cor triloculare biventriculare, is one of the rare congenital anomalies, (Garg et al., 2020) defined by a complete absence of the atrial septum, eventually associated with malformation of the atrioventricular (AV) valves. We report the case of a 57‐year‐old woman with CA complicated with Eisenmenger syndrome, who suffered from symptomatic persistent atrial fibrillation (AF). The patient did not have any muscular, skeletal, ophthalmologic, or vascular abnormalities to signify that her abnormalities were part of any congenital syndrome. Visceroatrial, atrioventricular, and ventriculoarterial concordance were noted. Nevertheless, she also presented inferior vena cava (IVC) interruption. During her last clinical evaluation, a mild holosystolic murmur was heard at all auscultation points, gaining intensity at the apex and mesocardiac area. On physical examination, while S1 was normal, a widely split S2 was heard. There was no pretibial edema, and the lungs were clear. Oxygen saturation measured with finger pulse oximetry was 92%.

**FIGURE 1:**
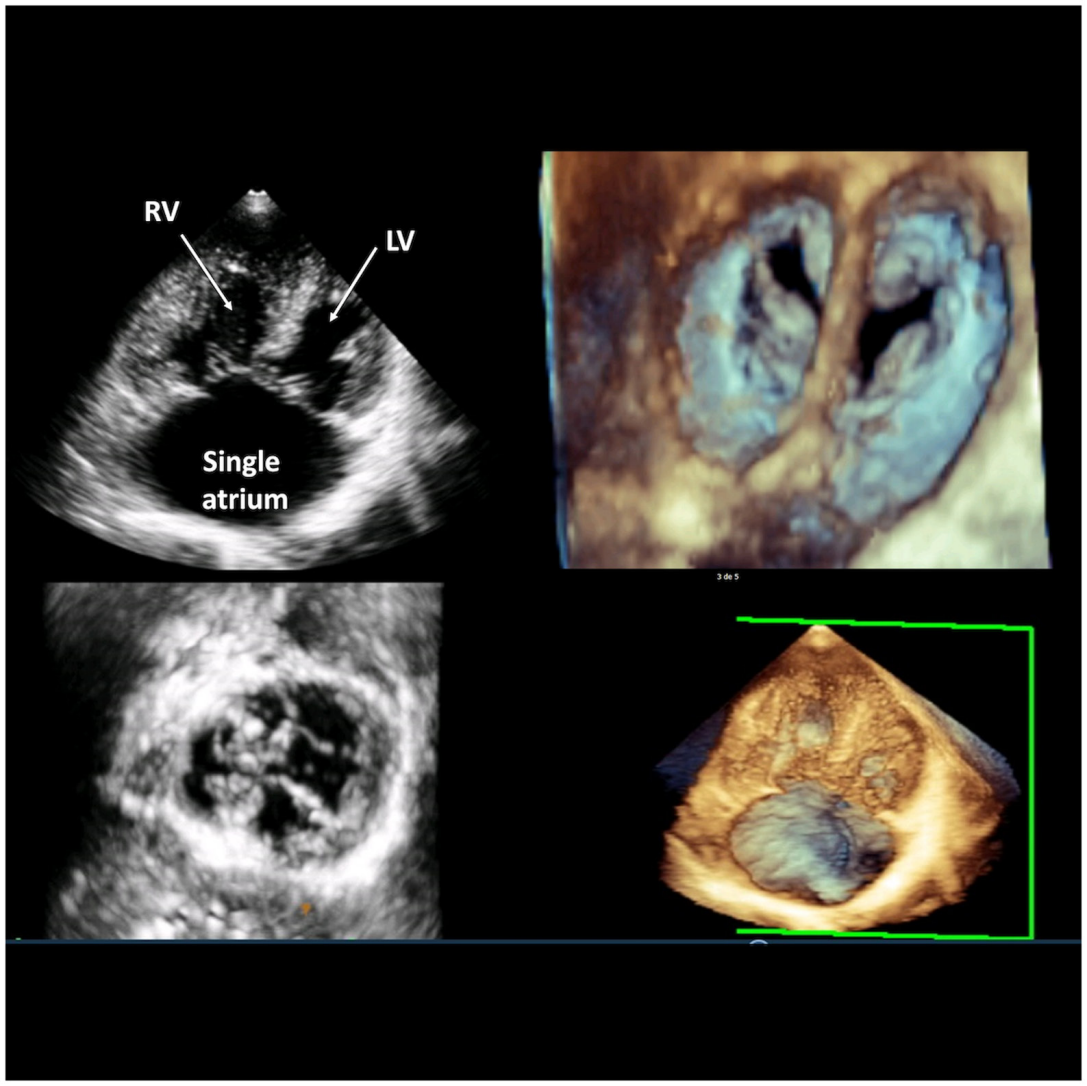
Two‐dimensional echocardiography images revealing common atrium/apical 4‐chamber view showing complete absence of interatrial septum. CA, common atrium; LV, left ventricle; RV, right ventricle.

Her last echocardiography evaluation showed in apical view the absence of interatrial septum (Figure 1). CA surface was 50 cm^2^. Left ventricular ejection fraction was 60%. In addition to right ventricular dilatation, moderate tricuspid regurgitation was seen, along with severe pulmonary hypertension. Tricuspid annular plane systolic excursion 17 mm. Mitral and tricuspid valve attachments to the interventricular septum were on the same anatomic plane, and there was a small cleft present in the mitral valve, with mild regurgitation. Paradox movement of the interventricular septum was observed but without any interventricular communication.

Cardiac catheterization revealed systolic pulmonary arterial pressure around 102 mmHg. Right ventricular pressure was 116/0 mmHg denoting severe pulmonary hypertension, RV end‐diastolic pressure at 21 mmHg, mean CA pressure as 13 mmHg, and left ventricular end‐diastolic pressure as 12 mmHg.

The patient was treated with furosemide, spironolactone, with anticoagulation by rivaroxaban. Rate control was achieved with a low dose of acebutalol. Because CA was diagnosed at a late stage, complicated with severe pulmonary hypertension despite combined therapy with bosentan and tadalafil (the later added since 2017), the patient was managed conservatively with no intervention to correct the congenital anomalies.

In March 2021, the patient presented a first episode of highly symptomatic persistent AF, with fast ventricular response (mean 150 beats per minute, with left anterior fascicular block, QRS width 110 ms) that benefited from successful electrical cardioversion (200 J delivered). Nevertheless, because of recurrent palpitations despite amiodarone after electrical cardioversion, a percutaneous AF ablation was decided 1 month later.

### 1.1 Atrial fibrillation ablation procedure

**FIGURE 2:**
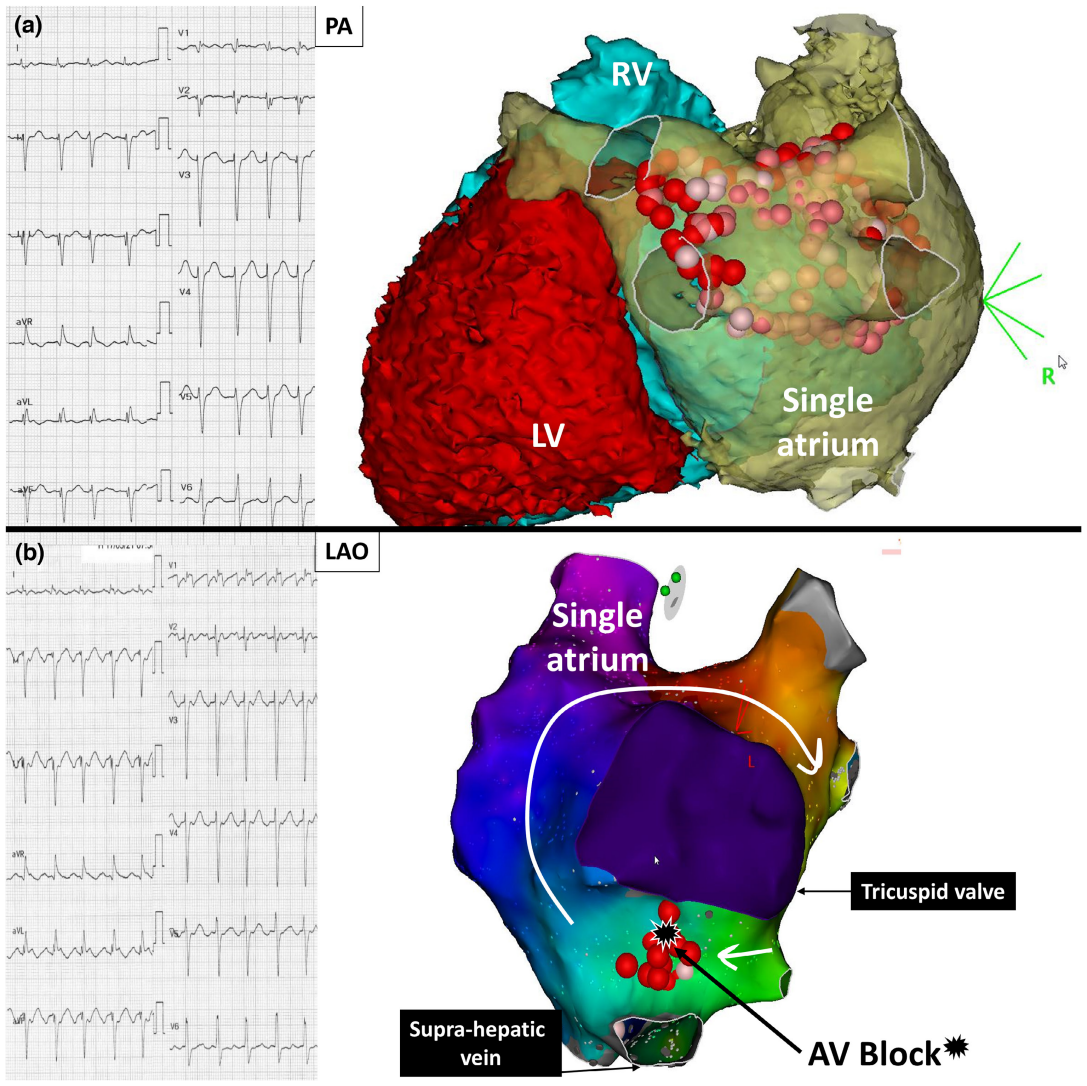
(a) Twelve‐lead electrocardiogram during atrial fibrillation and three‐dimensional electroanatomical map showing the ablation lesions around the pulmonary veins, and the posterior line. (b) Twelve‐lead electrocardiogram showing “typical” clockwise atrial flutter with 2:1 conduction, and the corresponding three‐dimensional activation map. An ablation line was created between the supra‐hepatic vein and the valve. The black star denotes the spot where complete atrioventricular block occurred. CA, common atrium; LV, left ventricle; RV, right ventricle.

Before planning the procedure, the patient underwent a computed tomography aiming for three‐dimensional reconstruction. The right superior vena cava was present and joined preferentially the right side of the CA. There was a connection of a supra‐hepatic vein at the inferior part of the CA, instead of the IVC. There was a normal drainage of pulmonary veins to the left side of the CA. The dilated right ventricle had a diameter of 56 mm, versus 47 mm for left ventricle. Pulmonary artery trunk diameter was measured at 46 mm. The patient was in sinus rhythm at the beginning of the procedure. Under mild sedation, an ultrasound‐guided puncture was performed, and three catheters were inserted via the left internal jugular vein: (Errahmouni et al., 2014) a decapolar catheter was inserted in the coronary sinus (CS; IBI®, Irvine, Abbott Medical); a multipolar catheter (PentaRay®, Biosense Webster), and a 3.5‐mm irrigated contact‐force‐sensing ablation catheter (SmartTouch ST‐SF®, Biosense‐Webster Inc.) were positioned in the CA. Calibration of contact force and respiratory gating were performed. Intravenous heparin (100 IU/kg) was administered immediately after femoral sheaths introduction and continuously infused (ACT > 350 s). After three‐dimensional geometry left atrium reconstruction (Carto System®, Biosense Webster Inc.), point‐by‐point RF delivery was performed aiming for a contiguous circular enclosing of the veins. Real‐time automated display of RF applications (Visitag®, Biosense Webster Inc.) was used with predefined settings for catheter stability (3 mm for 4 s) and minimum contact force (30% of time > 3 g). RF was delivered (SmartAblate®, Biosense Webster Inc.) in a power‐controlled mode with 35 to 45 W. Target values of ablation index were 450 to 550 (≥450) at the anterior wall, ≥400 on the roof and 300 to 400 (≥300) at the posterior wall. At the vicinity of the esophagus, RF was delivered until an ablation index target of 300. To achieve PVI, an inter‐vein line was performed within the right circle, associated with a posterior line as well (Figure 2). Complete PVI was achieved at the end of the procedure. The day after the procedure, a mild pericardial effusion was noticed, needing symptomatic treatment with colchicine. On day 3, the patient developed “typical” clockwise atrial flutter with prolonged QTc interval (600 ms), leading to amiodarone interruption, then switched by acebutalol. Because of persistent symptomatic atrial flutter (cycle length 270 ms) in the blanking period, another ablation procedure was performed 1 month later (Figure 2b). Activation mapping showed a peri‐valvular circuit that was interrupted by creating a line between the interrupted IVC and the valve. During RF delivery, an inadvertent and complete AV block occurred with junctional escape at 50 beats per minute, without any His potential recorded during high‐density mapping. A dual‐chamber pacemaker had to be implanted in the right pectoral region. The hospitalization was uneventful, and the patient was discharged after 2 days. After 20 months of follow‐up, the patient was free from any atrial arrhythmia recurrence without antiarrhythmic drug therapy.

## 2 DISCUSSION

To the best of our knowledge, this is the first description of PVI in a patient with CA. Since its first description by Young & Robinson (1907/1908), single atrium (SA) or CA was initially thought to be a form of endocardial cushion defect. Later, Levy et al. (1974) individualized SA as a specific entity, by reporting a case of complete absence of the atrial septum without any endocardial cushion defect. The authors recommended that the term SA should be reserved to the following condition: (a) complete absence of interatrial septum, (b) absence of malformation of the AV valves, and (c) absence of interventricular communication. Only three previous case reports described the presence of documented AF in patients with CA. The eldest patient to date with CA, was a 60‐year‐old female patient with permanent AF (Rawala et al., 2019). Kim et al. (2016) presented a 48‐year‐old male patient who was admitted for intermittent palpitations starting 1 week ago. AF was the initial manifestation leading to unmask CA. AF was managed with amiodarone and warfarin until surgery in this patient. In the case report from Altıntaş et al. (2017), AF was also the first manifestation of SA in a 43‐year‐old female patient, anticoagulated with dabigatran 150 mg bid. Actually, structural abnormalities (as well as genetic) should be investigated when discovering AF in young patients (Stoyanov et al., 2014).

Our case emphasizes the difficulties of performing an ablation procedure in complex anatomies. The first description of cavotricuspid isthmus ablation via a superior approach was reported by Varma et al. (2004). Later remote magnetic navigation was used to access transhepatic route via the femoral vein (Latcu et al., 2012). Even if associated with a high success in experienced centers, electrophysiological procedures are still challenging in the presence of IVC interruption (Al‐Sinan et al., 2022; Pons et al., 2012). In this recent study, IVC interruption was integrated with congenital heart disease in 43% of the cases. Liang et al. (2020) recently reported their experience of performing transseptal puncture for AF ablation via a superior access in case of interrupted or absent inferior vena cava. They identified 15 patients through a period from 2010 to 2019 who benefited from transseptal puncture via a superior access, but assisted with RF. They found that the procedures were successfully performed in these patients, but at the price of an increased prolongation of total procedural time, with associated significant increase in fluoroscopy exposure (57.0 ± 28.5 min; Liang et al., 2020).

Conduction disturbances are not rare in patients with endocardial cushion defects (Mehta et al., 1982). Hasanin & Kinsara (2008) reported the case of a 24‐year‐old soldier with syncopal attacks attributed to paroxysmal complete AV block in the setting of SA with persistent LSVC. Catheter ablation may be difficult in patients with congenital heart disease, because of potentially deviant and often unpredictable sites of the specific conduction system (Waldmann et al., 2022). Khairy et al. (2007) reported a case of inverted AV nodal input in a 25‐year‐old patient with surgically repaired partial AV canal defect.

## 3 CONCLUSION

A rare case of atrial fibrillation ablation is presented in a 57‐year‐old patient with common atrium and inferior vena cava interruption. Special considerations should be taken when performing an ablation procedure in this challenging anatomy, especially concerning the likelihood of atypical location of the atrioventricular node.

## AUTHOR CONTRIBUTIONS

S.‐S.B.: writing, review and editing; F.S. and D.S.: investigation and data collecting; P.M.: validation; E.F.: supervision.

## CONFLICT OF INTEREST STATEMENT

Nothing to disclose.

## ETHICS STATEMENT

The patient provided her consent for the study.

## Supporting information


Video S1

## Data Availability

The data that support the findings of this study are available from the corresponding author upon reasonable request.
